# Thioredoxin mitigates radiation-induced hematopoietic stem cell injury in mice

**DOI:** 10.1186/s13287-017-0711-2

**Published:** 2017-11-15

**Authors:** Pasupathi Sundaramoorthy, Qinhong Wang, Zhihong Zheng, Yiqun Jiao, Benny J. Chen, Phuong L. Doan, Nelson J. Chao, Yubin Kang

**Affiliations:** 1 Division of Hematological Malignancies and Cellular Therapy, Duke University Medical Center, DUMC 3961, 2400 Pratt Street, Suite 9000, Durham, NC 27710 USA; 2 Department of Hematology, Fujian Provincial Key Laboratory of Hematology, Fujian Medical University Union Hospital, Fuzhou, Fujian 350001 China

**Keywords:** Thioredoxin, Radiation exposure, Mitigation, Hematopoietic stem cells, Senescence

## Abstract

**Background:**

Radiation exposure poses a significant threat to public health. Hematopoietic injury is one of the major manifestations of acute radiation sickness. Protection and/or mitigation of hematopoietic stem cells (HSCs) from radiation injury is an important goal in the development of medical countermeasure agents (MCM). We recently identified thioredoxin (TXN) as a novel molecule that has marked protective and proliferative effects on HSCs. In the current study, we investigated the effectiveness of TXN in rescuing mice from a lethal dose of total body radiation (TBI) and in enhancing hematopoietic reconstitution following a lethal dose of irradiation.

**Methods:**

We used in-vivo and in-vitro methods to understand the biological and molecular mechanisms of TXN on radiation mitigation. BABL/c mice were used for the survival study and a flow cytometer was used to quantify the HSC population and cell senescence. A hematology analyzer was used for the peripheral blood cell count, including white blood cells (WBCs), red blood cells (RBCs), hemoglobin, and platelets. Colony forming unit (CFU) assay was used to study the colongenic function of HSCs. Hematoxylin and eosin staining was used to determine the bone marrow cellularity. Senescence-associated β-galactosidase assay was used for cell senescence. Western blot analysis was used to evaluate the DNA damage and senescence protein expression. Immunofluorescence staining was used to measure the expression of γ-H2AX foci for DNA damage.

**Results:**

We found that administration of TXN 24 h following irradiation significantly mitigates BALB/c mice from TBI-induced death: 70% of TXN-treated mice survived, whereas only 25% of saline-treated mice survived. TXN administration led to enhanced recovery of peripheral blood cell counts, bone marrow cellularity, and HSC population as measured by c-Kit^+^Sca-1^+^Lin^–^ (KSL) cells, SLAM + KSL cells and CFUs. TXN treatment reduced cell senescence and radiation-induced double-strand DNA breaks in both murine bone marrow lineage-negative (Lin^–^) cells and primary fibroblasts. Furthermore, TXN decreased the expression of p16 and phosphorylated p38. Our data suggest that TXN modulates diverse cellular processes of HSCs.

**Conclusions:**

Administration of TXN 24 h following irradiation mitigates radiation-induced lethality. To the best of our knowledge, this is the first report demonstrating that TXN reduces radiation-induced lethality. TXN shows potential utility in the mitigation of radiation-induced hematopoietic injury.

## Background

Radiation and radioactive substances are used extensively in medical research, disease diagnosis, and cancer treatment. Incidental radiation exposure could result from medical and radiological accidents, malfunction or breakdown of nuclear power plants, or a terrorist attack with radioactive dirty bombs. The danger of radiation injury is real, particularly in the aftermath of the 9/11 attacks in the US. Radiation injury impacts public health and society significantly. Between 1945 and 1987, there were 285 nuclear reactor accidents, injuring 1550 people and killing 64 [1–5]. These nuclear and radiological emergencies require comprehensive medical preparedness and readiness, including a national stockpile of deliverable agents to counteract radiation exposure incidents and accidents.

Hematopoietic stem cells (HSCs) and hematopoiesis are among the tissues/organs most sensitive to radiation injury and contribute to many of the manifestations of acute radiation injury, including bleeding, infection, and bone marrow failure. Evidence has emerged that excessive reactive oxygen species (ROS) following radiation injury cause HSC apoptosis and senescence and the loss of long-term repopulating capacity [6–9]. However, what remains poorly understood is how to harness the redox pathway for the mitigation of radiation-induced HSC injury. N-acetyl-l-cysteine (NAC) and glutathione have been shown to be able to protect HSCs from oxidative stress and promote the recovery of hematopoiesis when administered before radiation or immediately following radiation exposure. However, these compounds need to be given frequently (at least daily) and in large quantities (at least 100 mg/kg in mice) [10, 11]. Hematopoietic growth factors (HGFs) such as erythropoietin, thrombopoietin, and granulocyte-colony stimulating factor (G-CSF) have been tested in radiation injury for enhancing hematopoietic recovery [12, 13]. However, HGFs have limitations and drawbacks; these factors are lineage-specific and therefore do not promote the recovery of other cell lineages. Additionally, the efficacy of these growth factors is limited; G-CSF only quickens neutrophil recovery by a few days, and works only with a low dose of radiation exposure [11, 14–16]. Thrombopoietin needs to be administered soon after radiation exposure, and its efficacy is significantly limited if given 24 h after radiation exposure [17]. Currently, there is an urgent need to identify and develop novel agents that can be used to reduce radiation-induced HSC injury and enhance all lineage hematopoietic recovery when given 24 h after radiation exposure.

Using a semiquantitative, mass spectrometry-based proteomic approach, we recently screened for proteins that were differentially expressed in the bone marrow supernatants from hematopoietic stem cell transplant recipient mice that were treated with AMD3100 (a specific and reversible CXCR4 antagonist) [18]. We identified thioredoxin (TXN) as a novel molecule that has marked protective and proliferative effects on HSCs. TXN is a ubiquitous oxidoreductase with a molecular weight of 12 kDa and has two Cys residues in the conserved active site sequence (-Cys32-Gly-Pro-Cys35-). The primary function of TXN is to maintain redox homeostasis and to protect proteins from oxidative damage or inactivation [19–21]. We have shown that ex-vivo culture of murine HSCs with TXN or giving TXN to HSC transplant recipient mice enhanced the recovery and the long-term repopulation capacity of HSCs in our mouse models of HSC transplant [18]. However, the therapeutic potential of TXN as a radiation protectant or mitigator has not been investigated.

In the current study, we aimed to determine the effects of TXN in mitigating radiation-induced HSC injury when given 24 h after radiation exposure. Twenty-four hours after radiation has been chosen as a critical time point for evaluating the efficacy of any given agent as a radiation mitigator because, in the event of a radiation mass casualty scenario, a significant majority of patients will not present for therapeutic intervention for several hours following radiation exposure. To the best of our knowledge, this is the first report showing TXN to be an important agent for rescuing radiation-induced hematopoietic injury.

## Methods

### Mice

Eight- to 12-week-old female BALB/c mice were purchased from Jackson Laboratories (Bar Harbor, ME, USA) and used in this study. The mice were housed in our specific pathogen-free facility and maintained at 23–25 °C with a 12 h day/12 h dark cycle throughout the study, and were provided with autoclaved food and acidified water. All our studies were performed in accordance with Duke University Institutional Animal Care and Use Committee approved procedures.

### Cell culture

All the cell lines were purchased from American Type Culture Collection (Manassa, VA, USA). Primary fibroblast cells were grown in RPMI 1640 medium. The medium was supplemented with 10% fetal bovine serum (FBS) and 1% penicillin/streptomycin. Cells were maintained at 37 °C with 5% CO_2_ in a humidified incubator.

Murine lineage-negative (Lin^–^) cells were isolated from bone marrow cells (BMCs) using a lineage negative selection column as per the manufacturer’s instruction (Miltenyi Biotec Inc., Auburn, CA, USA). Briefly, BMCs were harvested from bilateral femurs and tibias of BALB/c mice and depleted of red blood cells (RBCs) using ACK lysis buffer (Lonza, Walkersville, MD, USA). Lin^–^ cells were then enriched using lineage negative selection columns. Isolated Lin^–^ cells were grown with 20 ng/ml mouse thrombopoietin (TPO), 125 ng/ml mouse stem cell factor (SCF), and 50 ng/ml mouse Flt3 ligand in 10% FBS with IMDM medium. Cells were maintained at 37 °C with 5% CO_2_ in a humidified incubator.

### Reagents and antibodies

Recombinant human thioredoxin-1 (TXN; *E. coli*-derived) was purchased from R&D Systems Inc. (Minneapolis, MN, USA). PE-conjugated anti-mouse Scal-1 and PerCP-5.5 lineage cocktail antibody was obtained from BD Biosciences (San Jose, CA, USA). FITC-conjugated anti-mouse CD48 and APC-conjugated anti-mouse CD 150 were obtained from Biolegend (San Diego, CA, USA). PE-Cyanine 7-conjugated anti-mouse CD117 (c-Kit) was purchased from eBioscience Inc. (San Diego, CA, USA). Senescence β-galactosidase (SA-gal) staining kit, γ-H2AX, p38 mitogen-activated protein kinase (MAPK), phospho-p38 MAPK (Thr180/Tyr182), p16, β-actin, and the corresponding secondary antibodies were purchased from Cell Signaling Technology (Danvers, Massachusetts, USA).

### Radiation injury mouse model and TXN treatment

BALB/c mice were irradiated with 7.25 Gy by total body irradiation (TBI) using a ^137^Cesium gamma irradiator (JL Shepherd, Glendale, CA, USA) at a dose rate of 4.18 Gy/min [22]. Mice were irradiated on a rotating platform. Twenty-four hours later, the mice were injected via the tail vein with TXN (1.6 mg/kg) every other day for a total of five doses, and the control group was administered with phosphate-buffered saline (PBS). Animal survival was monitored daily up to 30 days. In a separate sets of experiments, blood samples and BMCs were collected at 3 weeks or 6 weeks after radiation from surviving mice. Peripheral blood cell counts were measured by hematology analyzer. BMCs from femurs and tibias of the mice were measured for HSC population and cell senescence by flow cytometry and colony forming units (CFU).

### Peripheral blood analysis

Blood was directly withdrawn from the maxillary vein and collected in pre-coded EDTA-containing vials at 3 and 6 weeks following radiation exposure from surviving mice. Blood was mixed gently on a rotary shaker until analysis for white blood cells (WBCs), hemoglobin, and platelets on a hematology analyzer (scil Vet ABC Plus™, Gurnee, IL, USA).

### Flow cytometry analysis

To determine the percentage and absolute number of c-Kit^+^Sca-1^+^Lin^–^ (KSL) and SLAM + KSL cells after radiation from surviving mice, the mice were sacrificed and bones (2 femurs and 2 tibias per mouse) were harvested as described previously [18]. The cells were stained with PE-Cy7-CD117 (c-Kit), PE-Sca-1, and PerCP-5.5 lineage antibody, FITC-CD48, and APC-CD150 for 30 min for KSL and SLAM + KSL cell percentage. Acquisition was carried out using a BD-Canto II flow cytometer with the FACSDiva software (Becton Dickinson, San Jose, CA, USA).

### CFU assay

CFU assays were performed in complete M3434 methylcellulose medium (Stem Cell Technologies, USA) following the manufacturer’s instructions. Briefly, BMCs were mixed in complete M3434 medium and plated in 30-mm petri dishes at 20,000 cells per dish. The assay was performed in triplicate and the number of colony forming units-granulocyte macrophage (CFU-GM) and colony forming units-granulocyte, erythroid, macrophage, megakaryocyte (CFU-GEMM) were counted at day 7 and day 12, respectively.

### Bone marrow histology examination

Femurs were fixed in 10% neutral-buffered formalin. Specimens were then decalcified, embedded in paraffin, cut into 5-μm section, and stained with hematoxylin and eosin (H&E). The slides were examined by light microscopy to capture bright-field images using an Olympus (IX51) microscope (Japan).

### SA-gal activity analysis

To determine β-gal + senescent bone marrow cells, bone marrow was harvested and Lin^–^ cells were isolated by magnetic column purification using a mouse lineage cell depletion kit (Miltenyi Biotec) as described previously [18]. SA-gal activity in Lin^–^ cells was determined using a SA-gal staining kit from Cell Signaling Technology (Beverly, MA, USA) according to the manufacturer’s instructions.

Primary fibroblast cells were seeded into six-well plates at 1 × 10^4^ cells per well and incubated in a humidified atmosphere with 5% CO_2_ at 37 °C overnight. Cells were then irradiated with 3 Gy or 5 Gy and treated with TXN 10 μg/ml. Three days after irradiation, the cells were fixed and stained with the SA-gal staining kit as per the manufacturer’s instructions.

### Western blot

Cells were harvested, washed with PBS, and re-suspended in lysis buffer containing 50 mM Tris-HCl pH 7.4, 150 mM NaCl, 1 mM EDTA, 1% Triton × 100, 1% sodium deoxycholate, and 0.1% SDS. The cells were further lysed by brief sonication. The lysates were centrifuged at high speed for 10 min to remove the cell debris. Total protein was quantified using the DC protein estimation kit (Bio Rad) with bovine serum albumin (BSA) as a standard. Approximately 20 μg protein was loaded and run on SDS-PAGE. The proteins were transferred onto a nitrocellulose membrane. The membrane was blocked with 5% milk in Tris-buffered saline containing 0.1% Tween 20 (TBST), and primary antibodies were applied with 5% BSA in TBST overnight at 4 °C with gentle shaking. The membrane was then probed with HRP-conjugated secondary antibody and developed using the Pierce ECL substrate.

### Immunofluorescence

Primary fibroblast cells were irradiated (3 Gy) and cultured with and without TXN for 24 h. Cells were fixed in 100% cold ethanol for 10 min at –20 °C, permeabilized with Triton × 100 and washed with PBS. After blocking, cells were incubated overnight at 4 °C with anti-γH2AX rabbit antibody. The excess of unbound antibody was removed at each step by three washes with PBS. The cells were counterstained with DAPI. The images were captured on an Olympus confocal microscope (FV1000MPE, Olympus, Tokyo, Japan).

### Statistical analysis

All the data are presented as the mean ± SD. Comparisons were performed either by the student *t* test for analysis of variance for continuous data or by log-rank test for survival data. All statistical analyses were performed using Star View software (SAS institute, Cary, NC, USA) or Microsoft Excel (Microsoft, Seattle, WA, USA). *P* values less than 0.05 were considered significant.

## Results

### TXN rescues mice from a lethal dose of total body irradiation even when administered 24 h after irradiation

**Fig. 1:**
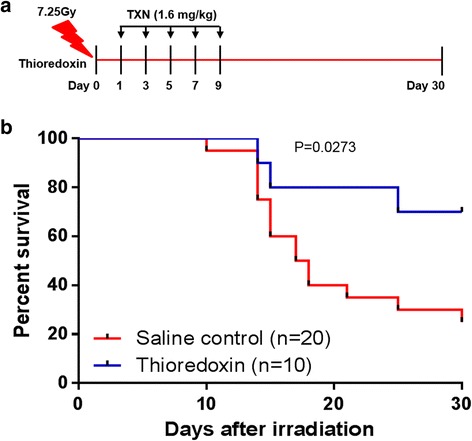
TXN mitigates TBI-induced lethality in mice. **a** Radiation and thioredoxin (*TXN*) treatment scheme for mice. BABL/c mice were irradiated with 7.25 Gy, and 1.6 mg/kg of TXN was administrated intravenously by tail vein at 24 h after irradiation and then every other day for a total of five doses. **b** The protective effects of TXN. Kaplan-Meier survival curve depicts the 30 day survival (*n* = 20 for saline-treated, lethally irradiated mice, and *n* = 10 for TXN-treated, lethally irradiated mice). One of two separate sets of experiments is shown

TXN has two major functions. First, TXN serves as one of the major antioxidants in mammals and protects cells from oxidative stress. Second, TXN is a cell growth factor and can modulate and stimulate diverse cellular processes by directly interacting with redox-sensitive or ROS-independent molecular pathways [20, 21]. TXN is an excellent candidate for drug development because of its structural stability, its ability to cross the cell membrane, and its ubiquitous expression. Previously, we found that TXN protected C57BL/6 mice from radiation-induced hematological injury and death when given 2 h after radiation exposure [18]. To test whether the protective effect of recombinant TXN can be generalized to other strains of mice and if TXN is still effective when given at 24 h after irradiation, BALB/c mice were total body irradiated with 7.25 Gy. Twenty-four hours later, the mice were given intravenous PBS control buffer or TXN at 32 μg per mouse (1.6 mg/kg body weight). The treatment was continued every other day for a total of five doses (Fig. 1a). The mouse survival was observed for 30 days. As shown in Fig. 1b, Kaplan-Meier analysis of survival indicated that TXN rescued mice from a lethal dose of radiation: 70% of TXN treated-mice survived the radiation whereas only 25% of saline-treated mice survived (*p* = 0.0273). These results suggested that TXN mitigates a lethal dose of TBI even when administered 24 h after radiation exposure.

### TXN enhances the reconstitution of murine hematopoietic stem cells after a lethal dose of total body irradiation

**Fig. 2:**
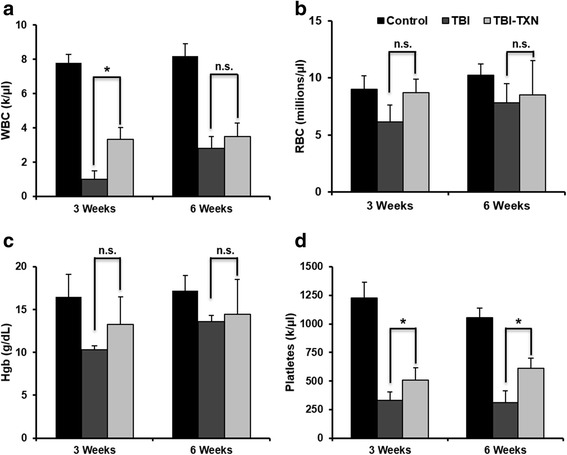
TXN mitigates TBI-induced pancytopenia in mice. Mice were irradiated with 7.25 Gy and then treated with saline or thioredoxin (*TXN*) as described in the text. The numbers of **a** white blood cells (*WBC*), **b** red blood cells (*RBC*), **c** hemoglobin (*Hgb*), and **d** platelets in peripheral blood were quantified at 3 and 6 weeks after total body irradiation (*TBI*). Data are shown as the mean ± SD. *P < 0.05. *n.s.* not significant

Radiation induces HSC damage and affects all lineages of blood cells. Protecting and rescuing HSCs from radiation damage are important in the treatment of radiation injury [8, 23]. Therefore, we sought to investigate the in-vivo effect of treatment with TXN on hematopoietic cells. BALB/c mice were irradiated with 7.25 Gy followed by TXN administration 24 h later as described in Fig. 1a. Peripheral blood cell counts were measured at 3 weeks and 6 weeks after radiation in surviving mice. As shown in Fig. 2a, WBC counts were significantly increased in TXN-treated mice at 3 weeks following radiation in comparison to saline-treated mice (*p* < 0.05). The levels of RBCs and hemoglobin were not significantly changed by TXN treatment, although there was a trend for higher numbers of RBCs and hemoglobin in TXN-treated mice at 3 weeks following radiation (Fig. 2b and c). The platelet levels were significantly increased at both 3 and 6 weeks (*p* < 0.05) by TXN administration (Fig. 2d). These data indicate a faster hematological recovery of WBCs and platelets following TXN treatment after radiation exposure.

**Fig. 3:**
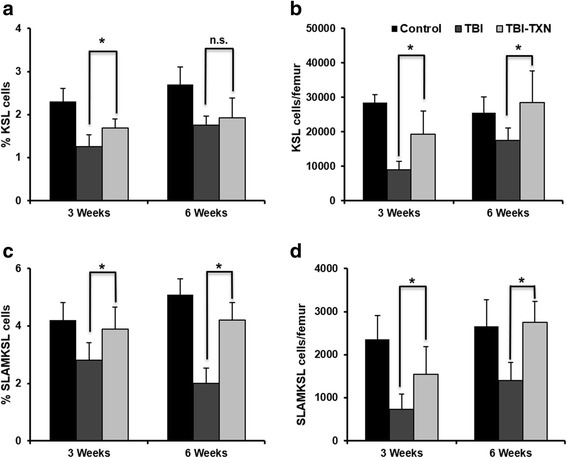
TXN mitigates TBI-induced suppression of HSCs in mice. Mice were irradiated with 7.25 Gy and then treated with saline or thioredoxin (*TXN*) as described in the text. The frequency of HSCs in bone marrow was analyzed by flow cytometry; **a** percentage of KSL cells and **b** the absolute number of KSL cells/femur; **c** percentage of SLAM + KSL (*SLAMKSL*) and **d** the absolute number of SLAM + KSL cells/femur. Data are shown as the mean ± SD. *P < 0.05. *KSL* c-Kit^+^Sca-1^+^Lin^–^, *n.s.* not significant, *TBI* total body irradiation

We next analyzed the bone marrow HSC population at 3 weeks and 6 weeks after radiation. We measured the percentage and the absolute number per femur of bone marrow KSL cells and SLAM ^+^ KSL cells using FACS analysis. KSL cells are a mixed population of murine hematopoietic stem cells and hematopoietic progenitor cells. SLAM ^+^ KSL cells represent primitive, long-term repopulating hematopoietic stem cells [24]. We found that TXN given at 24 h after irradiation significantly increased the numbers of KSL cells (Fig. 3a) (*p* < 0.05) and SLAM ^+^ KSL cells (Fig. 3b) (*p* < 0.05).

**Fig. 4:**
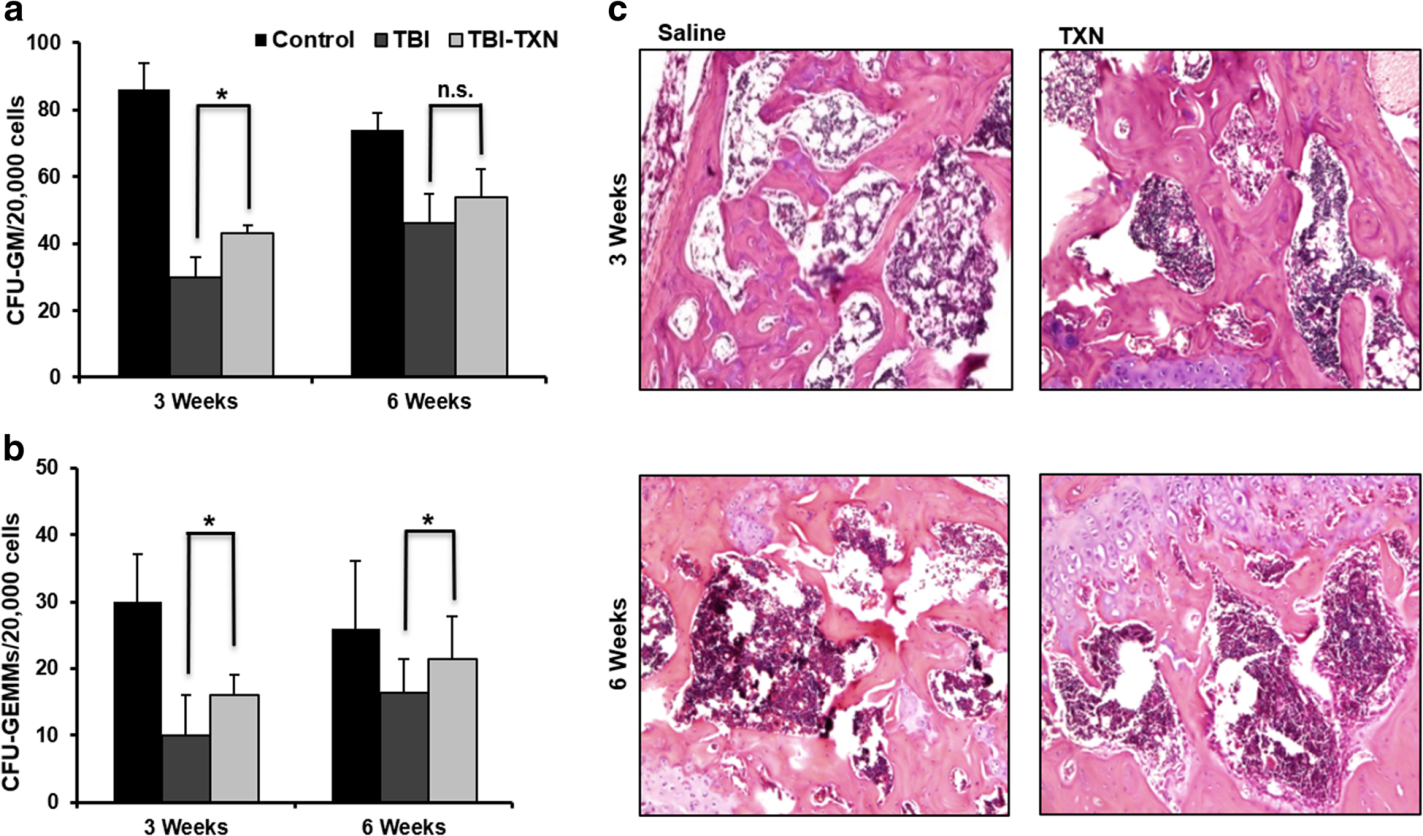
TXN facilitates expansion of hematopoietic progenitor cells and increases bone marrow cellularity. The effects of thioredoxin (TXN) on the colongenic function of HSCs in bone marrow were measured by CFU assay in total body irradiation (*TBI*) mice for **a** colony forming unit-granulocyte macrophage (*CFU-GM*) and **b** colony forming unit-granulocyte, erythroid, macrophage, megakaryocyte (*CFU-GEMM*). **c** Effects of TXN on bone marrow cellularity in TBI mice. Panels show H&E staining of mouse femurs. Representative images are shown for saline and TXN treatments. Data are shown as the mean ± SD. *P < 0.05. *n.s.* not significant

The numbers of colony forming units (CFUs) serve as an indicator for hematopoiesis and an important sign for hematopoietic recovery. We thus measured bone marrow CFUs in TXN-treated mice and saline-treated mice at 3 weeks and 6 weeks after lethal irradiation. We found that TXN administration significantly increased CFU-GM and CFU-GEMM (Fig. 4a and b) (*p* < 0.05). Radiation-induced bone marrow damage resulted in massive ablation of the cellular content in the bone marrow, and decreased bone marrow nucleated cells (BMNCs) [23]. We thus examined the bone marrow cellular content by H&E staining and found that TXN treatment reduced the radiation-induced cellular depletion and increased bone marrow cellularity (Fig. 4c). These data suggest that TXN mitigates the TBI-induced bone marrow damage by enhancing hematopoiesis and facilitating stem cell regeneration to accelerate the hematopoietic recovery.

### TXN reduces cell senescence after a lethal dose of TBI

**Fig. 5:**
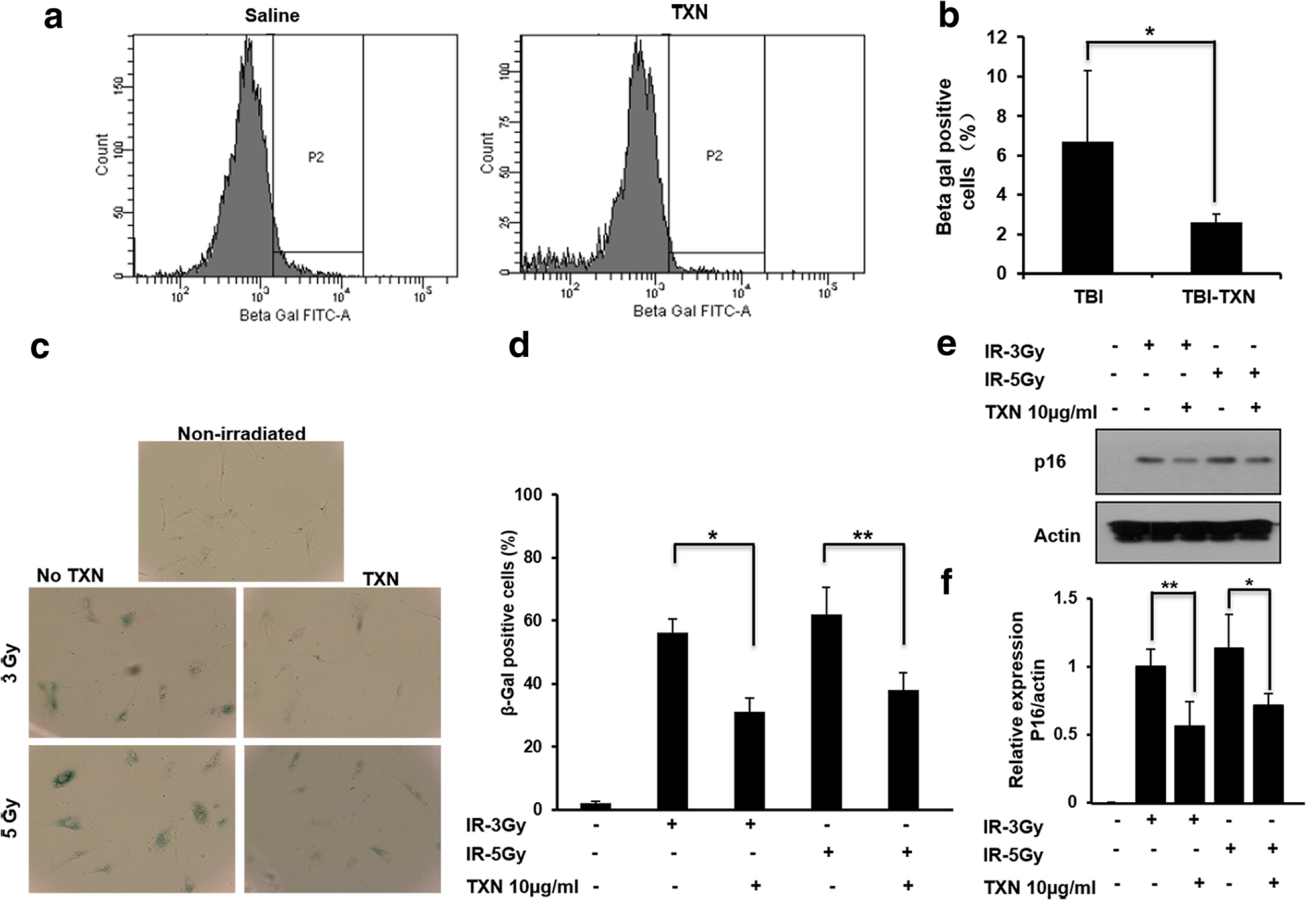
TXN mitigates TBI-induced cell senescence in vivo and in vitro. Mice were irradiated with 7.25 Gy and then treated with saline or thioredoxin (*TXN*) as described in the text. **a** At 3 weeks after radiation, the Lin^–^ cells were isolated and stained with β-galactosidase, analyzed by FACS and **b** quantified. **c** Primary fibroblast cell lines were irradiated with 3 and 5 Gy and cultured with and without TXN for 3 days and then stained with β-galactosidase kit. Cells were photographed under a light microscope (magnification, 200×) and **d** β-gal-positive cells were quantified. **e** The levels of p16 protein expression were analyzed by Western blot and **f** quantitative densitometry of the protein expressions. Data are shown as the mean ± SD. *P < 0.05; **P < 0.01. *IR* ionizing radiation, *n.s.* not significant, *TBI* total body irradiation

Cell senescence occurs after exposure to radiation and is one of the major biological processes that leads to the impairment of HSC function and the loss of HSC self-renewal capacity [7, 25]. Therefore, we examined the effects of TXN on radiation-induced cell senescence in vitro and in vivo. BALB/c mice were irradiated and treated with TXN or saline as described in Fig. 1a. At 3 weeks after radiation, bone marrow Lin^–^ cells were isolated from the mice and stained for senescence-associated β-galactosidase (SA-gal) activities. TXN significantly reduced radiation-induced cell senescence in murine bone marrow Lin^–^ cells by FACS analysis (Fig. 5a and b).

To further confirm the effects of TXN in mitigating radiation-induced cell senescence, we evaluated the TXN effect in vitro using primary fibroblasts. We irradiated primary fibroblasts with 3 Gy or 5 Gy and cultured the cells with or without TXN for 72 h. We chose 3 Gy and 5 Gy because 3 Gy was found to cause hematopoietic damage symptoms [1] and 5 Gy could result in death in 50% of exposed individuals from the sequelae of hematopoietic damage unless there is medical intervention [2]. As shown in Fig. 5c and d, irradiation induced cell senescence as demonstrated by increased SA-gal-positive cells after irradiation. TXN treatment significantly reduced SA-gal-positive primary fibroblasts, indicating that TXN treatment reduced radiation-induced cell senescence in primary fibroblasts.

p16 (cyclin-dependent kinase inhibitor 2A) has been involved in the establishment and maintenance of cellular senescence and is an important marker of cellular senescence [25, 26]. Consistent with SA-gal activity, the expression of p16 level was reduced with TXN treatment (Fig. 5e and f). Collectively, these data suggest that TXN treatment rescues cells from radiation-induced cell senescence.

**Fig. 6:**
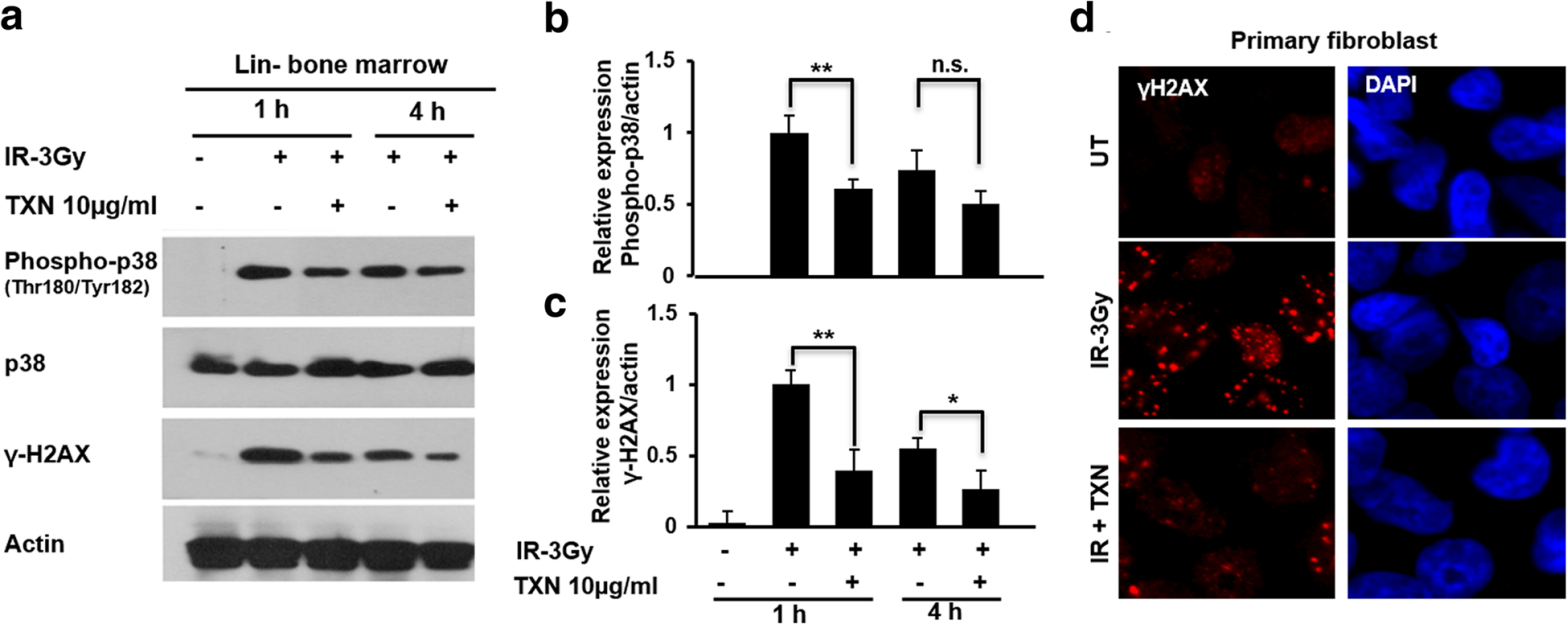
TXN reduced γH2AX and p38 expression after TBI. Lin^–^ BMCs were isolated and culture with appropriate growth factor as described in the text. Lin^–^ BMCs were irradiated with 3 Gy, and treated with and without thioredoxin (*TXN*) for 1 and 4 h. **a** Western blot analysis of p38, phospho-p38, γH2AX, and GAPDH protein expression. Quantitative densitometry of the protein expressions of **b** phospho-p38 and **c** γH2AX. **d** Primary fibroblasts were irradiated with 3 Gy with and without TXN for 1 h and stained with γH2AX foci (*red*) and DAPI (*blue*). Data are shown as the mean ± SD. *P < 0.05; **P < 0.01. n.s., no statistical difference. *IR* ionizing radiation, *n.s.* not significant, *UT* untreated

Studies have found that p38 MAPK is activated after radiation exposure and the activated p38 mediates cell senescence, apoptosis, and loss of self-renewal capacity in HSCs [27–29]. To determine the effects of TXN on p38 in murine hematopoietic cells, murine bone marrow cells were enriched for hematopoietic stem/progenitor cells using a lineage-deletion column. Murine Lin^–^ cells were irradiated and treated with TXN. Phosphorylated p38 (Thr180/Tyr182) and total p38 levels were measured at 1 and 4 h after irradiation. TXN treatment downregulated radiation-induced phosphorylated-p38 (Thr180/Tyr182) in murine Lin^–^ bone marrow cells (Fig. 6a and b).

### TXN reduces radiation-induced DNA double-strand breaks

DNA double-strand break is a hallmark of radiation damage. Histone H2AX becomes phosphorylated on serine 139, called γ-H2AX, as a reaction to DNA double-strand breaks. γ-H2AX has been used as a marker for DNA double-strand breaks [30, 31]. To test if TXN could reduce DNA double-strand breaks and/or enhance DNA repair, we measured the effects of TXN on γ-H2AX. Murine Lin^–^ BMCs were irradiated with 3 Gy and treated with and without TXN for 1 and 4 h. We found that TXN treatment led to a reduced level of γ-H2AX expression after radiation (Fig. 6a and c). Additionally, we measured γ-H2AX foci in primary fibroblast cells (Fig. 6d). Consistent with the results in murine Lin^–^ cells, TXN significantly reduced the level of γ-H2AX in primary fibroblasts.

## Discussion

Accidental radiation exposure or a terrorist attack with a radioactive dirty bomb poses a serious threat to public health. Management of radiation injuries is a complex medical challenge, requiring a careful encounter as well as therapeutic agents administered at the appropriate time following the radiation exposure [1, 23, 32]. HSCs and hematopoiesis are among the tissues/organs most vulnerable to radiation injury. Radiation-induced damage to HSCs leads to HSC cell senescence and defects in HSC self-renewal capacity, and contributes to several manifestations of acute radiation sickness. Currently, there are very few agents that can effectively rescue HSCs from radiation injury when given after radiation exposure [10]. Antioxidant and HSC growth factors have limitations, and the major drawback of these agents is that they should be administrated before radiation or immediately after radiation [33–35]. There is an unmet medical need for identifying and developing effective agents that can be used to rescue lethal-dose radiation injury and enhance all-lineage hematopoietic cell recovery when given after irradiation.

Previously, we reported that TXN mitigates mice from radiation-induced death and enhances HSC recovery when given at 2 h after radiation exposure in a mouse model of radiation injury [18]. Our current study has important clinical relevance. Individuals exposed to radiation may not be aware of the exposure until a few hours later. Therefore, agents that are effective in rescuing victims from radiation injury when given 24 h after radiation exposure have great potential for clinical use. The remarkable ability of TXN to mitigate radiation-induced damage when administered intravenously 24 h after lethal TBI makes it an attractive radiation countermeasure agent for further development in the use against radiation injury. Further supporting this proposition is that TXN is highly competent in ameliorating the radiation-induced hematopoietic injury by facilitating HSC recovery. We have shown that TXN enhanced the recovery of multi-lineages of peripheral blood cells such as WBCs and platelets. TXN-treated mice demonstrated more cellular bone marrow. Importantly, TXN treatment had a higher number of CFU-GEMM, KSL cells, and primitive SLAM ^+^ KSL HSCs.

Radiation causes HSC damage through several mechanisms: increased production of ROS and induction of oxidative stress [36, 37]; increased oxidative DNA damage [38, 39]; activation of apoptotic cell death [40]; enhanced cell senescence [41, 42]; and promotion of HSC differentiation [43]. Cell senescence, an irreversible proliferative arrest, plays a critical role in radiation-induced HSC injury. HSC senescence impairs HSC replication and self-renewal, and thus reduces the HSC long-term repopulating capacity [44]. Originally described as an antioxidant, TXN also plays numerous roles as a transcription factor and signaling molecule [20, 21]. The levels of TXN were found to correlate with organismal lifespan [45, 46]. Pharmacological and genetic inhibition of TXN induced premature senescence in skin fibroblasts and hepatic cancer cells, suggesting a role for TXN in the regulation of cell senescence [47, 48]. Our results demonstrated that TXN-treated mice had lower SA-gal-positive cells compared to saline-treated mice. The effects of TXN in reducing cell senescence after radiation injury were further validated in primary fibroblasts. p16 is one of the vital biomarkers and an important mediator for cell senescence [27, 49]. TXN treatment suppressed the p16 expression level, further supporting the effects of TXN on cell senescence.

Activation of the p38 pathway contributes to the induction of p16 and HSC senescence following exposure to irradiation [43, 50]. It has been shown that radiation causes HSC cell senescence through the activation of the p38 pathway, and the inhibition of p38 activity with a specific inhibitor (SB203580) attenuated radiation-induced hematopoietic cell injury. Inhibition of p38 activity appears to be a promising strategy for HSC proliferation. We have shown that TXN is able to downregulate phosphorylated p38. TXN serves as a potential mediator of redox signaling by ROS-dependent and -independent pathways [20, 51] and is involved in the regulation of multiple biological processes such as antiapoptotic, anti-inflammatory, and mitogenic activities [20]. The major target of TXN in the cytosol is apoptosis signal-regulating kinase 1 (ASK1). ASK1 is a member of MAP3 kinase family, which activates both the c-Jun N-terminal kinase (JNK) and p38 MAPK pathways [52]. TXN binds to ASK1 and prevents ASK1 from full activation, thus downregulating the p38 pathway [29].

Radiation-induced DNA damage can occur due to the direct effect of radiation on DNA molecules, which accounts for 30–40% of lesions, or by free radicals, which accounts for 60–70% of lesions [39, 53]. Irradiation induces a variety of DNA lesions, including oxidized base damage, abasic sites, single-strand breaks (SSBs), double-strand breaks (DSBs), and DNA protein crosslinks [54]. DSBs are thought to be the most lethal lesion induced by irradiation, as one unrepaired DSB can be sufficient to trigger apoptosis [8, 17, 22]. γH2AX is a vital marker for DSBs. We have found that TXN reduces γH2AX expression in both murine Lin^–^ bone marrow cells and in primary fibroblasts. The reduction in γH2AX expression could be due to less double-strand DNA breaks from its antioxidant function and/or enhanced DNA repair by TXN. Our preliminary data indicate that TXN could upregulate the gene expression of the Fanconi anemia/BRCA DNA repair pathway (data not shown).

Radiation damage is complex and there are many mechanisms underlying radiation damage such as iNOS and cytokines [24], miRNA regulation [55], NF-kB activation [56], caspase-dependent apoptosis [8], and LC-II-induced autophagy [57]. In addition to reducing cell senescence and downregulating p38 and γH2AX as shown in the current study, TXN likely acts on other signaling pathways and affects various cellular events. TXN can act as a cellular growth factor and promotes the proliferation of B cells and various transformed cells [58, 59]. Since ERK1/2 and JNK are members of the MAPK family, TXN modulation on ERK1/2 and JNK after radiation cannot be excluded. Further studies with these two enzymatic molecules should be explored. Recent studies have implicated TXN in the regulation of cell cycle progression through G2/M [60] and in the p53-mediated base excision repair pathway [61]. TXN activates the MEKK1-JNK signaling pathway, leading to IkB degradation and NF-kB activation [56]. It has been shown that TXN directly interacts with PTEN, inhibits phosphatase activity and membrane binding of PTEN, and activates the Akt pathway [62]. TXN can translocate to the nucleus and regulates the functions of several transcription factors including Ref-1, GR, HSF1, HDAC4, HIF1a, NFkB, Nrf2, PPARg, RUNX2, and SP1 [63, 64].

In the current study, TXN was given intravenously every other day for five doses. We are currently optimizing thioredoxin administration regimens and testing different administration routes, including intramuscular or subcutaneous injection. Intramuscular or subcutaneous injection will offer a simpler and more practical route of administration, particularly in a mass casualty scenario. Importantly, TXN has several important features that make it an attractive candidate for further development as a radiation mitigator. TXN promotes the recovery of hematopoietic stem cells and enhances the recovery of multiple lineages of hematopoietic cells. This is significant as G-CSF only works on myeloid progenitors and only enhances the recovery of neutrophils. TXN affects and modulates diverse cellular events, including cell senescence, apoptosis, and double-strand DNA breaks. TXN is a ubiquitously expressed endogenous protein, eliminating the concerns of developing immune response following administration. TXN can cross the cell membrane and enter cells efficiently. Therefore, TXN can be simply added into an HSC culture or administered systematically.

## Conclusion

In summary, our study shows that TXN effectively mitigates TBI-induced hematopoietic injury in mice, even when given 24 h after radiation exposure. Our results demonstrate that TXN can be potentially used as an effective medical radiation countermeasure.

## Abbreviations

ASK1: Apoptosis signal-regulating kinase-1
BMC: Bone marrow cell
BSA: Bovine serum albumin
CFU-GEMM: Colony forming unit-granulocyte, erythroid, macrophage, megakaryocyte
CFU-GM: Colony forming unit-granulocyte macrophage
CFU: Colony forming units
DSB: Double-strand break
FBS: Fetal bovine serum
G-CSF: Granulocyte-colony stimulating factor
H&E: Hematoxylin and eosin
HGF: Hematopoietic growth factor
HSC: Hematopoietic stem cell
JNK: c-Jun N-terminal kinase
KSL: c-Kit^+^Sca-1^+^Lin^–^
MAPK: Mitogen-activated protein kinase
PBS: Phosphate-buffered saline
RBC: Red blood cell
ROS: Reactive oxygen species
SA-gal: Senescence-associated β-galactosidase
SSB: Single strand break
TBI: Total body irradiation
TXN: Thioredoxin
WBC: White blood cell
